# Extraction of Mechanical Parameters via Molecular Dynamics Simulation: Application to Polyimides

**DOI:** 10.3390/polym16060813

**Published:** 2024-03-14

**Authors:** Philipp Rosenauer, Christoph Kratzer, Silvia Larisegger, Stefan Radl

**Affiliations:** 1 Institute of Process and Particle Engineering, Graz University of Technology, Inffeldgasse 13/III, AT-8010 Graz, Austria; christoph.kratzer@student.tugraz.at (C.K.); radl@tugraz.at (S.R.); 2 KAI GmbH, Europastraße 8, AT-9524 Villach, Austria; silvia.larisegger@k-ai.at

**Keywords:** molecular dynamics simulations, polyimides, Young’s modulus, Poisson’s ratio

## Abstract

Polyimides feature a vast number of industrial applications due to their high thermal stability and insulation properties. These polymers exhibit an exceptional combination of thermal stability and mechanical toughness, which allows the semiconductor industry to use them as a mechanical stress buffer. Here, we perform all-atom molecular dynamics (MD) simulations for such materials to assess their predictive capability with respect to their mechanical properties. Specifically, we demonstrate that the OPLS-AA force field can be used to successfully describe an often-used polyimide (i.e., Kapton^®^) with respect to its Young’s modulus and Poisson’s ratio. Two different modes to extract these mechanical properties from MD simulations are presented. In particular, our continuous deformation mode simulations almost perfectly replicate the results from real-world experimental data and are in line with predictions using other MD force fields. Our thorough investigation of Kapton^®^ also includes an analysis of the anisotropy of normal stresses, as well as the effect of simulation properties on the predicted Young’s moduli. Furthermore, the polyimide pyromellitic dianhydride/2-(4-aminophenyl)-1H-benzimidazole-5-amine (PMDA-BIA) was investigated to draw a more thorough picture of the usability of the OPLS-AA force field for polyimides.

## 1. Introduction

Polyimides have a vast number of applications in the field of semiconductor protection. They are used as a passivation stress buffer to protect the semiconductor chip surfaces from thermal and mechanical stresses in packaging processes. Furthermore, they are used as a protective layer from α-rays released by inorganic fillers in epoxy-molding compounds (EMC) and as a final insulation layer before interconnect bumping operations. Adapting and improving their behavior in regard to mechanical and thermal protection behavior is an important topic in materials science. Combining both the molecular dynamics simulations with experimental characterizations is often beneficial (or even necessary) to obtain a deeper understanding of influencing factors [1,2,3].

The first step in an attempt to improve the properties of a polymer with simulation is to verify the accurate representation of the reality with the used simulation parameters. Therefore, physical properties are predicted by means of computer simulations that can easily be compared to experimental or literature data. Our group has already investigated the density and glass-transition temperature properties.

Lei et al. [4] investigated the thermo–mechanical properties of six different polyimide films, including PMDA-ODA (Poly [4,4′-oxydiphenylenepyromellitimide]). Kapton^®^ is the brand name of PMDA-ODA. In their work, they used the COMPASS force field and the so-called “Stress–Strain” scripting in the Material Studio simulation package. The Material Sstudio simulation package is a commercially available software tool that brings along licensing costs. Lei et al. also investigated the polymer PMDA-BIA, which was investigated as a second polyimide in our current work, as the literature data for it is extremely sparse.

Odegard et al. [5] predicted the mechanical properties of polymers with different force fields. In their study, they investigated a single polyimide (i.e., BPDA-APB) with the force fields AMBER, OPLS-AA, and MM3. The prediction of the mechanical properties, namely the Young’s modulus and the shear modulus, was most accurate in comparison to experimental values with the OPLS-AA force field. However, Kapton^®^ was not investigated by them, and we placed the focus of this work on determining the quality of predicted mechanical properties of Kapton^®^ with the OPLS-AA force field.

Riccardi et al. [6,7] described a method to determine a locally resolved Young’s modulus and Poisson’s ratio. In their work, they investigated an atactic polystyrene (PS) thin film and a polystyrene nanocomposite. For describing the atactic polystyrene, the OPLS-AA (“optimized potentials for liquid simulations all-atom”) [8] force field was used. PS has a comparably simple structure to simulate in comparison to the objective polymers of the present study, i.e., polyimides.

The objective of our present study is to determine the applicability of the OPLS-AA force field to the polyimides (i) Kapton^®^ (or PMDA-ODA) and (ii) pyromellitic dianhydride/2-(4-aminophenyl)-1H-benzimidazole-5-amine(PMDA-BIA). In order to determine if the mentioned force field can be applied to PMDA-ODA and PMDA-BIA, (i) a thorough investigation of how to determine the Young’s modulus and the Poisson’s ratio was conducted, and (ii) the computed results were compared to the literature data. Kapton^®^ was used as it is a commercially available standard polymer. PMDA-BIA was used as it is a similar polymer with different properties. In future research, we plan to use Kapton^®^ in combination with an aqueous liquid phase. Therefore, the OPLS-AA force field was used.

The Young’s modulus and Poisson’s ratio were determined via molecular dynamics with either (i) a simulation of the well-established tensile test [9,10,11,12] or (ii) the methodology proposed by Riccardi et al. [6,7]. To allow for more flexibility, contrary to previous works ([4]), in this work, the large-scale atomic/molecular massively parallel simulator (LAMMPS) [13] was used with the OPLS-AA force field.

The first section of this work describes the simulation and modeling details to allow the interested reader to replicate our results. Specifically, the preparation procedure for the virtual polymer molecules and the numerical parameters used by the software are described. Additionally, the different procedures used to calculate Young’s modulus will be described.

The results section is organized in a way that the effect of each influencing parameter, like the system size, temperature, and pressure, are described individually for each deformation mode. This allows the reader to understand the different application fields of the deformation modes and what parameters to look for. The last part of the result section compares the behavior of the material in the simulations with literature and experimental data.

## 2. Materials and Methods

### 2.1. Molecular Dynamics Simulation Method

The molecular dynamics simulations in the present study were conducted with LAMMPS [13] (LAMMPS branch: stable; commit: 584943fc928351bc29f41a132aee3586e0a2286a (28 October 2020)). To allow for the greatest flexibility in polymers that are investigated, the base geometry of a monomer is determined by vector coordinates in an xyz-file. The atomic structure of the monomers that were investigated in our present work were extracted in the form of bond lengths and angles from Ramos et al. [14]. With the atomistic bond lengths and angles, it was possible to calculate the coordinates of each atom of the monomer to prepare the xyz-file. Moltemplate [15] (git branch: master; commit: 9f1512e6b25f8325b5c6c1a4f2fecdc94eda40f1 (21 March 2021)) was used to transform this xyz-file into the required LAMMPS input file collection. Moltemplate allows the user to determine the size of the simulation box, the chain length, and the number of chains, which is beneficial for the investigation of polymeric materials.

As the initial state for a molecular dynamics simulation is created artificially, the system needs to be equilibrated before the actual simulation runs. The equilibration removes structural artifacts that occurred during the initialization of the system [16] and imposes the correct pressure and temperature on the system for the simulation. There are various ways and definitions to ensure that an equilibrated state has been reached [16]. In this study, the system was equilibrated based on the 21-step equilibration that uses a certain procedure of NVT (constant temperature, constant volume) and NPT (constant temperature, constant pressure) ensembles to reach that equilibrated state [17]. In the present study, the maximum pressure was set to be 50,000 (atm), and the final pressure and final temperature represent ambient conditions at 1 (atm) and 300 (K).

### 2.2. Simulation Parameters

Here, we describe the simulation parameters used in the present study to allow the reader the replication of the simulations. The described parameters were used for all systems of both investigated polymers.

Both for the relaxation mode and the continuous deformation mode, periodic boundaries were imposed. Atom style “full” was chosen with real units. Bond, angle, and improper style were harmonic, and the OPLS style was used for dihedrals. The styles determine how the bonded forces between all atoms of the polymer of interest are calculated. The formulas for these potentials can be found in the LAMMPS manual [13]. For non-bonded interactions, the pair style lj/cut/coul/long was used, which computes a standard 12/6 Lennard–Jones potential with an additional coulombic pairwise interaction. In LAMMPS, a long-range solver is used to compute long-range coulombic interactions. Most of these long-range solvers perform their computation in K-space, which we also prefer by using the “kspace style” command. As k-space style “pppm” with an accuracy of 0.0001 is used, and “kspace modified gewald” with a rinv value of 0.0002 [Å^−1^] is used to ensure that every simulation is conducted with the same solver conditions. Here, rinv represents the G-ewald parameter for Coulombics and is given in reciprocal distance units.

### 2.3. Force Field and Numerical Parameters

The “optimized potentials for liquid simulations” (OPLS) force field was used in its all-atom form to describe the interatomic interactions. This force field was developed by Jorgensen et al. [18,19] and is widely used in molecular dynamics simulations [20]. Additionally, moltemplate has the OPLS force field already included, which allows for a less time-intensive preparation of the polymer system.

### 2.4. Chain Length Selection

The chain length selection impacts the behavior and results of the molecular dynamics simulation [21,22]. The system behaves stiffer if the chain length is longer, i.e., the systems tend to have a stiffer elastic regime and higher yield stresses [22]. In particular, the entanglement is influenced by the chain length and is shown in the yield peak. Shorter chains can more easily align with the direction of loading and show a less pronounced yield peak. However, computational power is the limiting factor in molecular dynamics simulation; therefore, a chain length of 20 monomers was chosen as a compromise. This was motivated by the fact that model chains with 10–20 repeating units were previously found to be sufficient for reasonable results [23,24,25,26] without inducing size effects.

For reasons of comparability, the chain length that was chosen for Kapton© was also used for the simulations involving the PMDA-BIA polyimide.

### 2.5. System Size

The system size in molecular dynamics simulations is an important metric both for the results of the simulation and the required computational power. An important task is to find the sweet spot between those criteria and get the most representative results with the least possible computational power. Before the final system sizes were defined, some preliminary simulations were conducted to compare three system sizes. Table 1 shows the size of the different systems, the number of chains in each system, and the chain length. The preliminary results showed that system A was not behaving isotropically already in our preliminary analysis. Furthermore, although Ries et al. [27] showed that the system size has no impact on their results, their systems were all larger than 104 atoms. Due to these reasons, we decided to remove system A from the investigation but kept the naming, as displayed in Table 1.

Investigations of system size were skipped from the beginning for the additionally investigated polymer PMDA-BIA, as it was assumed that the smallest system would not produce usable results. As the number of atoms per monomer group is different for the two systems, Table 2 shows the respective number of atoms for each system.

### 2.6. Relaxation Mode

This method was first introduced by D. N. Theodorou and U. W. Suter [9] to predict the elastic constant of a glassy polymer. Further development of the procedure was conducted by Riccardi et al. [6,7] to locally resolve Poisson’s ratio and Young’s modulus of composite materials. The main idea behind this method is to apply a small deformation at time t0 and let the system relax afterwards. The relation of stress, strain, and elastic stiffness coefficient matrix are related according to Equation (1). Under the assumption of an isotropic material, this relation can be reduced to Equation (2):

(1) $$\bar{\sigma }=\overset{̿}{C}\cdot \bar{\varepsilon }$$

(2) $$\left[\begin{array}{c} \varepsilon_{xx}\\ \varepsilon_{yy}\\ \varepsilon_{zz} \end{array}\right]=\frac{1}{E}\;\left[\begin{array}{ccc} 1 & -\nu & -\nu \\ -\nu & 1 & -\nu \\ -\nu & -\nu & 1 \end{array}\right]\cdot \left[\begin{array}{c} \sigma_{xx}\\ \sigma_{yy}\\ \sigma_{zz} \end{array}\right].$$

**Figure 1:**
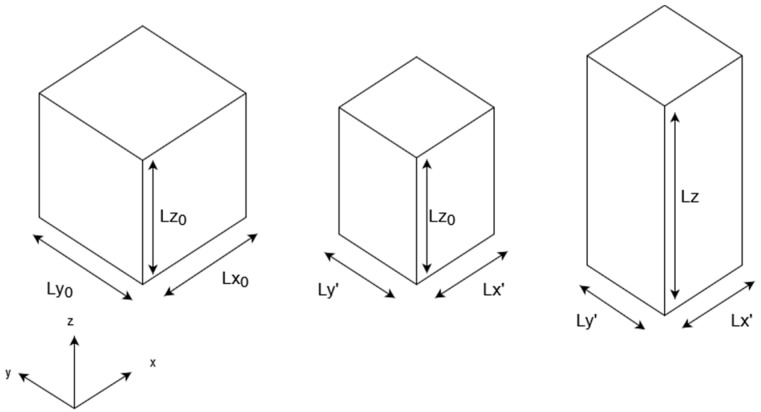
Depiction of the method to determine Young’s modulus and Poisson’s ratio via “relaxation mode” simulations.

The main benefit of the method is the ability to locally resolve Poisson’s ratio in a compound material. Therefore, the strain is imposed in two directions uniformly; otherwise, the local displacements would be a function of the overall material properties if periodic boundary conditions were used [6]. During the deformation, the length of the edges in the third axis is held constant. After inducing the strain, an equilibration phase follows, whereby the length in the deformed directions is held constant in contrast to the unperturbed axis, which can change its length to balance the emerging stress. Figure 1 shows the basic principle of this “relaxation mode”-type of simulation.

Due to the biaxial strain scheme imposed on the system in the x and y direction, *σ_xx_* = *σ_yy_* = *σ*_‖_, and *ε_xx_* = *ε_yy_* = *ε*_‖_. With the further assumption of mechanical equilibrium, the stress *σ_zz_* = 0; thus, Equation (2) results in two linear equations, i.e., Equations (3) and (4). By rearranging these equations, a direct measurement of Young’s modulus (Equation (5)) and Poisson’s ratio (Equation (6)) is possible. The same rearrangements could be made by inducing the strain as a combination of the other directions:

(3) $$\sigma_{∥\;}-\nu \sigma_{∥}=E\varepsilon_{∥}$$

(4) $$-2\nu \sigma_{∥}=E\varepsilon_{zz}$$

(5) $$E=\frac{2\sigma_{∥}}{2\varepsilon_{∥}-\varepsilon_{zz}}$$

(6) $$\nu =-\frac{\varepsilon_{zz}}{2\varepsilon_{∥}-\varepsilon_{zz}}$$

Due to fluctuations in the MD simulations, 100 computed values were combined to one averaged value to smoothen the results. Furthermore, the final value of Young’s modulus was determined by averaging the results of the last 50% of the simulation time. The simulation time in the relaxation mode experiments was 5 ns.

### 2.7. Continuous Deformation Mode

**Figure 2:**
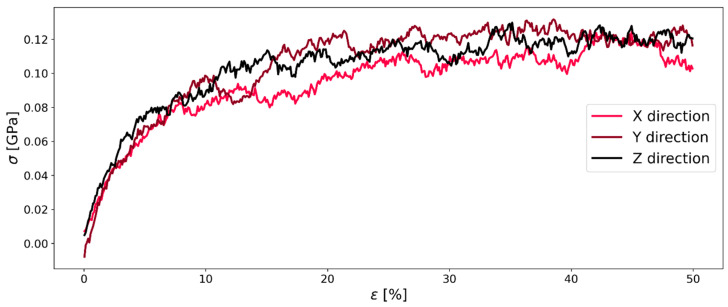
Stress–strain diagram of stretching system C of Kapton^®^ as shown in Table 1 with a strain rate of 1 × 10^−7^.

The continuous deformation mode represents the tension test that is well-known and widely used to determine Young’s modulus and Poisson’s ratio of elastic materials. In the conventional tension test, Young’s modulus is determined from the stress–strain curve with Equation (7), as shown in Figure 2. Here, ε is the deformation in percent of the initial length as shown in Equation (8), and σ is the stress in the normal stress in the material. Another important factor that is affecting the result of the simulation is the deformation rate $\dot{\varepsilon },$ defined in Equation (9) [28]:

(7) $$E_{i}=\frac{\sigma_{i}}{\varepsilon_{i}}$$

(8) $$\varepsilon =\frac{L-L_{0}}{L_{0}}$$

(9) $$\dot{\varepsilon }=\frac{∆\varepsilon }{∆t}$$

As mentioned previously, to ensure that each simulation starts at the same initial state, the system is equilibrated before the actual simulation run. In the next step, the system is deformed in one direction with a certain deformation rate up to a maximum deformation, depending on the simulation time. It is important to mention that the deformation rate is extremely high in comparison to the real tension experiments where the deformation rate is between $10^{-4}\left[s^{-1}\right]$ and $10^{5}\left[s^{-1}\right]$. In all our continuous deformation mode simulations of the present work, the rate is between $10^{-8}\left[fs^{-1}\right]$ and $10^{-5}\left[fs^{-1}\right]$, thus, $10^{2}$ to $10^{5}$ higher than the highest deformation rate of the real experiments.

The Young’s modulus can then be calculated as the slope of a straight line fit between the origin (at ambient pressure) and the respective stress, for which the Young’s modulus is calculated. There is not one Young’s modulus, but each strain has a different Young’s modulus as the slope of the straight-line fit varies with different strains. The anisotropy Γ and the linearity in the form of R^2^ were introduced to allow for an objective determination of Young’s modulus.

### 2.8. Anisotropy Γ

It is important that a small system (as modeled in an MD simulation) can represent the bulk of the polymer of interest as adequately as possible. One important property that needs to be represented is the isotropy. Therefore, every system was deformed in the three spatial directions *x*, *y*, and *z*. In the next step, normalized perpendicular stress was determined for each direction following Equations (10) and (11).

(10) $$\bar{\sigma }=\frac{\sigma_{x}+\sigma_{y}+\sigma_{z}}{3}$$

(11) $$\sigma_{i}^{*}{,}_{n}=\frac{\sigma_{i}}{\bar{\sigma }}$$

(12) $$\Gamma_{\left(\varepsilon \right)}=\frac{1}{N}\sum_{N=\varepsilon -0.01}^{N=\varepsilon }\sigma_{\sigma_{i}^{*}{{,}_{n}}_{\left(\varepsilon \right)}}\;$$

The anisotropy is calculated by averaging the standard deviation of the normalized perpendicular stress $\sigma_{\sigma_{i}^{*}{,}_{n_{\left(\varepsilon \right)}}}$ of the last percent of strain before the strain (corresponding to the value of Γ) is calculated (Equation (12)).

### 2.9. Linearity

The second determining factor is the R^2^ value of a straight line that is fitted to the simulation results to obtain Young’s modulus. Determining Young’s modulus in this way is only valid when the system follows Hooke’s law up to the first plastic deformation. Hooke’s law is valid when there is a linear relationship between the deformation and the stress at this deformation [29]. In order to verify the linearity quantitatively and, therefore, to probe the validity of Hooke’s law, the R^2^ value of the fit was logged. A high R^2^ represents a good representation of the results by the straight line fit.

### 2.10. Software

The software used for the simulation runs (i.e., LAMMPS), the pre-processing steps, and the exact version (i.e., commits) of the software was already mentioned at the beginning of the Methods Section (see “Molecular Dynamics Simulation Method”). The post-processing tools that were used for visualization were (i) Microsoft^®^ Excel^®^ for Microsoft 365 MSO (Version 2401 Build 16.0.17231.20236) 64-bit, and (ii) self-written python scripts (Python 3.10.9 64-bit.) developed in the Spyder IDE (version 5.4.1).

## 3. Results and Discussion

### 3.1. Effect of System Size and Isotropy

**Figure 3:**
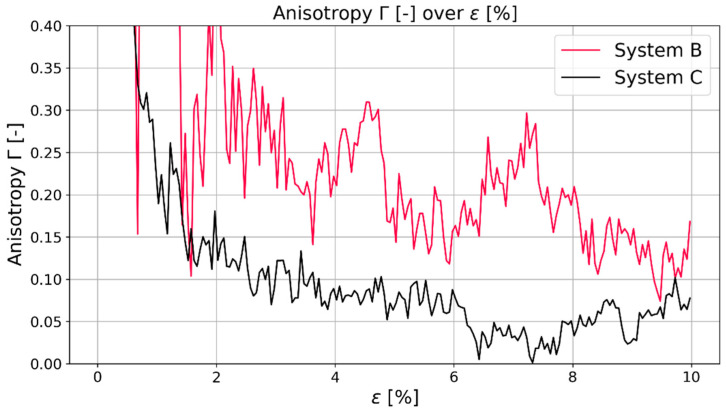
Comparison of anisotropy Γ of system B (red) and system C (black) for Kapton©.

The first thing that was investigated was the effect of the system size on the isotropy of the polymer system. As mentioned in the section about the relaxation mode, it is necessary for the system to be isotropic, i.e., to be able to use Equations (5) and (6) to calculate Young’s modulus and Poisson’s ratio. Both systems B and C were investigated regarding their isotropy. The isotropy was investigated based on the already described anisotropy Γ. Figure 3 shows the anisotropy in the first 10% of strain of system B and system C. From about 1.7% on, the black line that represents the anisotropy of system C is below the red line of system B. In this case, a lower anisotropy represents a more isotropic system.

**Figure 4:**
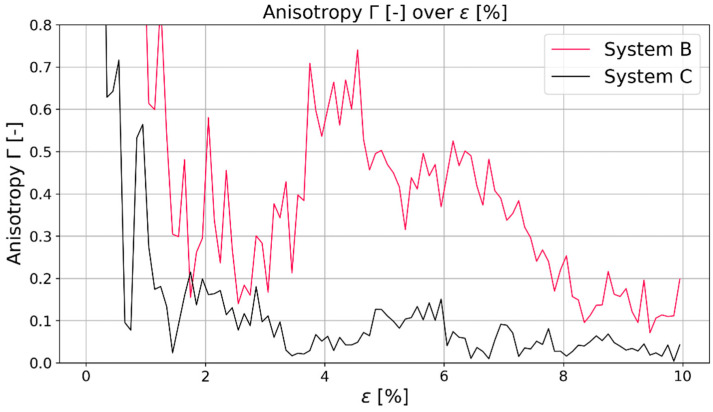
Comparison of anisotropy Γ of system B (red) and system C (black) for PMDA-BIA.

From this investigation, it can be seen that additional to the chain length mentioned by [21,22], the system size is also relevant for MD predictions of the polymeric system. Both systems have the same chain length, but system C has five times more chains in comparison to system B. Further differences that surfaced due to the size difference was the behavior during stretching and compressing: regarding the calculated Young’s moduli of system B and system C, the general trend was that the difference between the moduli in system B is about 20% between compression and stretching while the difference in system C was about 10%. Although simulations were conducted for both system sizes, the results presented in the next sections were taken from the simulations of system C, as there was a clear indication that the larger system leads to more representative results. Figure 4 shows the same behavior for the second investigated polymer PMDA-BIA. With the confirmation of this system size-dependent behavior, it can be said that bigger systems are beneficial for simulations that require a certain isotropy for representative results.

### 3.2. Effect of the Temperature

#### 3.2.1. Relaxation Mode

The results from the relaxation mode simulations represent the assumed behavior from the literature: Lyulin et al. [11] showed a linear decrease of the Young modulus with increasing temperatures up to the glass-transition temperature. Here, we also demonstrate that above the glass-transition temperature, Young’s modulus leveled off and stayed constant, although temperatures were increasing for both polymers. With rising temperatures, the Young’s modulus and the Poisson’s ratio were decreased. The investigation of Young’s modulus for Kapton^®^ over the temperature showed a change in slope between 600 (K) and 700 (K), which coincides with the glass-transition temperature described in the literature [30]. Contrary, for PMDA-BIA, there was a steep decline in Young’s modulus between 500 K and 600 K. This coincided with the found glass transition temperature of density monitoring from our simulations but did not coincide with the glass-transition temperature reported by Lei et al. [4].

#### 3.2.2. Continuous Deformation Mode

In the continuous deformation mode, the Young’s modulus also leveled off with rising temperature, but it was also seen that the behavior of the system changed with rising temperatures. For the equilibration process at 300 (K) the system started to expand when the simulation was conducted at higher temperatures. This led to a negative stress, although the system was stretched in one direction, and the stress should have been positive. Additionally, it was found that there was no linear behavior between higher deformation and stress, but the stress response stayed the same upon a continued deformation of the material. This leads to our speculation that around the glass-transition temperature, where this behavior change was the highest, the system lost parts of its tensile strength.

This behavior in the continuous deformation mode indicates that the equilibration method should be adapted before the measuring simulation. We assume that this can be done either (i) if the last NPT step of the 21-step equilibration is already conducted at elevated temperatures or (ii) an additional equilibration step is included before the measuring run to give the system the chance to equilibrate at the temperature of the simulation before measuring the stress–strain behavior. However, this would be a task for further investigation.

### 3.3. Effect of the Pressure

**Figure 5:**
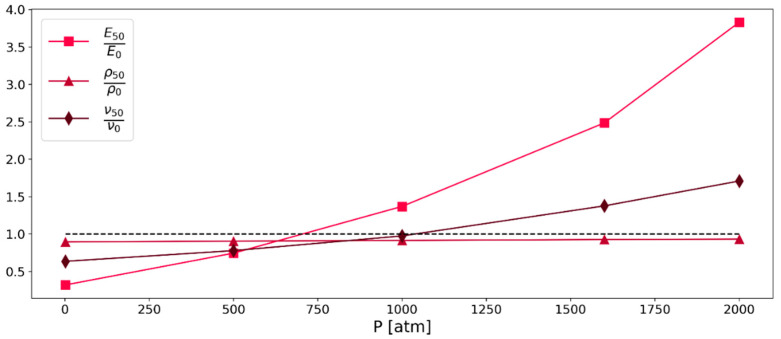
Pressure dependence of Young’s modulus, Poisson’s ratio, and density from relaxation mode simulations (system C) of Kapton^®^. The black dotted line represents the target value at 1 as this graph visualizes simulation values normalized to literature values.

**Figure 6:**
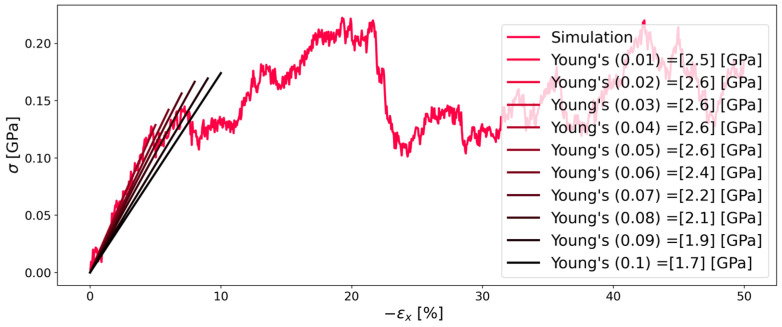
Stress–strain diagram of system C of Kapton^®^ during compression in the x-direction at 1 (atm). The simulation results are depicted as the red line. Straight lines with changing colors represent the linear fit for each corresponding strain (indicated in parenthesis in the legend).

**Figure 7:**
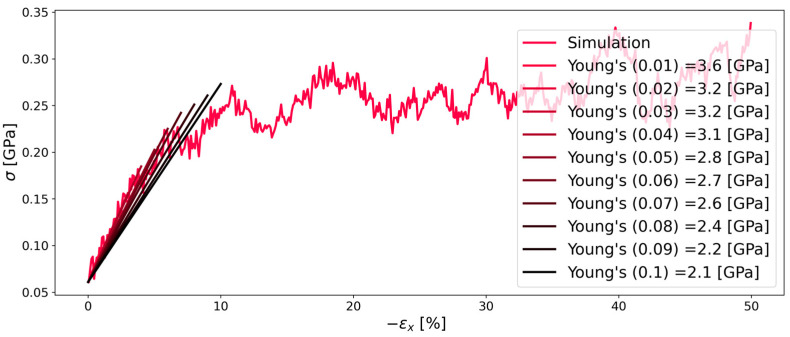
Stress–strain diagram of system C of Kapton^®^ during compression in the x-direction at 750 [atm]. For interpretation of the legend, see the description in Figure 6.

As the stress in molecular dynamics simulations is determined with the compute pressure command, it was assumed that the pressure will have a certain effect on the simulation results. In the MD simulations, the pressure was logged for each simulation run (i.e., stress and pressure were computed using LAMMPS’ “compute pressure” command). This was done in order to systematically investigate the effect of pressure on the system properties. Simulations regarding the Poisson’s ratio, Young’s modulus, and density were conducted in relaxation mode and normalized with the respective literature data. The results of these simulations are summarized in Figure 5. As can be seen in Figure 6 and Figure 7, the results of the continuous deformation mode also show a dependence on the pressure, but this effect is smaller than what is observed in the relaxation mode simulations.

#### 3.3.1. Relaxation Mode

For the determination of the best representation of the real behavior by the simulation, a normalized Young’s modulus, a normalized density, and a normalized Poisson’s ratio were used. The values were normalized with data from the literature [30]; therefore, the best result for each of the investigated values is 1. For the simulation results the latter half of the 5 ns simulation run was averaged for each pressure and normalized as mentioned above. The best fit was determined to be at 750 (atm). Based on these findings, it was decided that simulations of the continuous deformation mode should be conducted at 750 atm as well. Simulations at this pressure resulted in the best agreement with literature, in respect to Young’s modulus, density, and Poisson’s ratio, as shown in Figure 5.

#### 3.3.2. Continuous Deformation Mode

Figure 6 and Figure 7 show that also the continuous deformation mode simulations are affected by different pressures, but to a smaller extent, as a result of the relaxation mode simulations. In order to determine the Young’s modulus for each percent of deformation, a linear fit was applied to the simulation data. Specifically, as a least squares method was used, the linear fit had different ordinate distances that represented the stress at zero strain. For ambient pressure, this needs to be zero in an equilibrated system. Therefore, the linear fit was forced to pass through the origin. On the contrary, at a pressure of 750 (atm), the stress at zero strain was higher as the elevated pressure also induced a certain stress on the system, although there was no strain applied at the beginning of the simulation. For this case, the linear fit was forced to pass through the stress determined at zero strain for each percentage of deformation. Both simulations at 1 (atm) and 750 (atm) showed reasonable agreement for Young’s modulus when compared to the literature value of 2.5 (gPa) [30].

**Figure 8:**
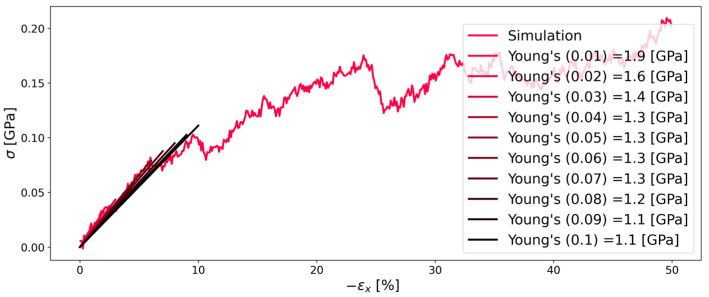
Stress–strain diagram of system C of PMDA-BIA during compression in the x-direction at 1 (atm). The simulation results are depicted as the red line. Straight lines with changing colors represent the linear fit for each corresponding strain (indicated in parenthesis in the legend).

**Figure 9:**
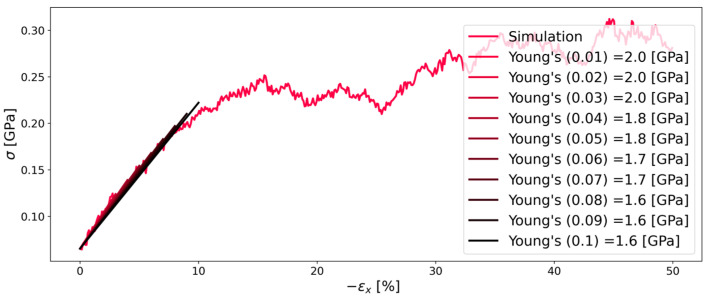
Stress–strain diagram of system C of PMDA-BIA during compression in the x-direction at 750 (atm). For interpretation of the legend, see description in Figure 8.

Based on the findings from the investigation of Kapton^®^, the second polymer PMDA-BIA was investigated at the same pressures and temperatures for reasons of comparability. Figure 8 and Figure 9 show that the behavior of both polymers is similar. Contrary to Kapton^®^, it is interesting that there was less influence of the pressure on the Young’s modulus for PMDA-BIA. Furthermore, there is very little literature data that allows a comparison of the Young’s modulus of PMDA-BIA: in contrast to the agreeing results of Kapton^®^, the simulated results of this study were far off the results by Lei H. et al. [4], which is the only literature data that we found on this polymer. As the data were in good agreement with the experimental data, and the same procedure as for Kapton^®^ was used, the reliability of the literature data was questioned. In [4], a Young’s modulus higher than 8.5 GPa was reported for PMDA-BIA, while the results of our study were in the order of 2 GPa.

### 3.4. Youngs Modulus and Poisson Ratio

**Figure 10:**
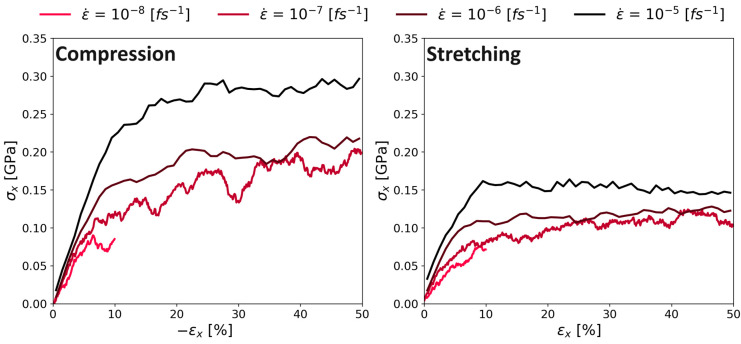
Stress–strain relation for different strain rates on system C of Kapton^®^. **Left** panel with induced compression; **right** panel with induced stretch.

The stress–strain curve during the continuous deformation mode for both compression and expansion is shown in Figure 10. Our results showed the same known behavior of higher stresses at higher strain rates as observed for other polymers in MD simulations [11,22,31]. There was no detectable strain softening after the yield point in compression and a plateauing of the stress in stretching. There was no strain softening regime after the yield point, which also represents the typical behavior of a polyimide.

The general behavior for all stress–strain curves during the continuous deformation mode stayed the same: First, there is a linear increase of the stress, followed by a region in which the slope decreases. Finally, the stress is almost constant (only for the slowest deformation rate 10^−8^ (fs^−1^); there is a significant temporal fluctuation of the stress) till the end of the simulation. Also, there is a clear difference in the performance of the compression and stretching of the system: By stretching the system, the maximum stress was below the results of the compression simulations, especially at high strain rates. We speculate the following: The entangled chains need some time to relax after deformation is initiated. During stretching, entangled chains become loose-packed, which leads to lower intermolecular forces and hence, stresses. In contrast, during our compressive deformation simulations, the chains increasingly entangle with ongoing deformation, building up extremely high stress (approximately twice as high as during stretching). As we will show later, this also leads to extremely high values of the Poisson’s ratio.

**Figure 11:**
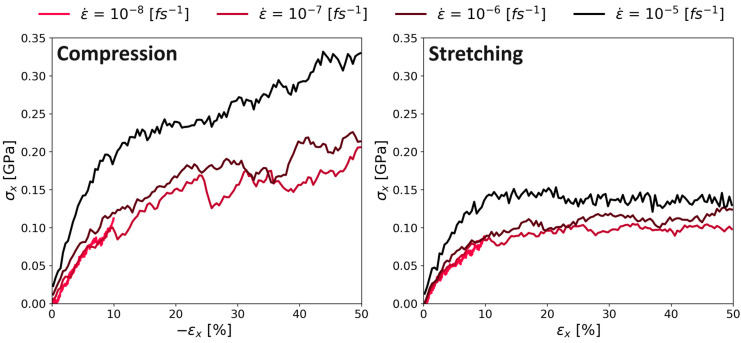
Stress–strain relation for different strain rates on system C of PMDA-BIA. Left panel with induced compression; right panel with induced stretch.

Figure 11 shows a similar behavior for PMDA-BIA. The stresses during compression (shown in the left panel) were higher than during stretching, as in the case of Kapton^®^. While the behavior during stretching was very similar to the stretching of Kapton^®^, the compression of PMDA-BIA at the lowest deformation rate kept a linear upward trend after the initial steep linear behavior.

**Figure 12:**
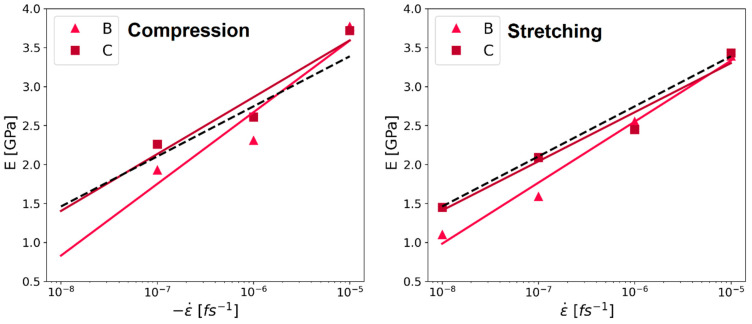
Young’s modulus of Kapton^®^ analyzed at 2% strain, versus strain rate, simulated values as squares for system C, and triangles for system B. The plotted lines are fits using the least square method. The colors of the lines replicate the colors of the symbols for the respective system sizes described in the legend. Left panel: compression. Right panel: stretching. Results are in good agreement with values found in experiments using Kapton^®^ HPP ST [33], indicated as a black dashed line.

A noticeable semi-logarithmic relationship between the Young’s modulus and the strain rate was observed in the literature [32,33]. Specifically, this behavior was observed in laboratory experiments that indicated that a faster deformation leads to a higher Young’s modulus. Exactly such a trend was also observed in our compression and stretching simulation data, as shown in Figure 12.

Analyzing the slope of regression lines from Young’s modulus versus strain rates results in a slope of 0.27 for the Kapton^®^ (system C). This result was similar to the results observed during experiments using HPP ST polyimide films with a slope of 0.28 [33]. Also, the other simulated values agree well with values between 0.34 and 0.23. However, the laboratory trials were performed at significantly slower strain rates between $10^{-4}$ and $10^{-1}$ $\left[s^{-1}\right].$ The coefficient of determination R^2^ in the compression simulations is 0.91 for system C and 0.90 for system B. In the case of the stretching deformation mode, the coefficients of determination are even higher (i.e., 0.97 for system C and 0.98 for system B).

Besides the similar slope to the existing literature, the increased Young’s modulus caused by longer chains matches the expected behavior, as well as results for different polymers during MD simulations (Refs. [11,22,31]; all these simulations used the continuous deformation mode).

**Figure 13:**
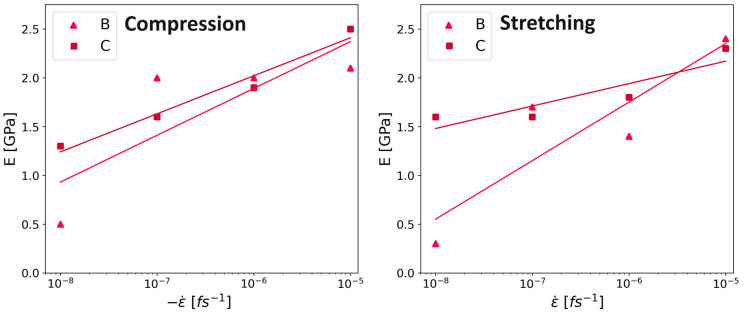
Young’s modulus of PMDA-BIA analyzed at 2% strain, versus strain rate, simulated values as squares for system C, and triangles for system B. The plotted lines are fits using the least square method. The colors of the lines replicate the colors of the symbols for the respective system sizes described in the legend. **Left** panel: compression. **Right** panel: stretching.

Although there were no values from experiments regarding the impact of the deformation rate on the Young’s modulus for PMDA-BIA, the same simulations as for Kapton^®^ were conducted to see if the same semi-logarithmic behavior could be found. Similarly to Kapton^®^, the Young’s modulus of system C of PMDA-BIA showed a semi-logarithmic behavior, as can be seen in Figure 13. In contrast, the behavior of system B could not be described by the semi-logarithmic fit for PMDA-BIA. The coefficient of determination for compression for system C was 0.91, and for system B it was 0.44. In the stretching simulations, the coefficient of determination for system C was 0.98 and for system B was 0.75. This is another indication that the system size is relevant, and that a larger system is beneficial for the simulation results of molecular dynamics simulations conducted in this study.

**Figure 14:**
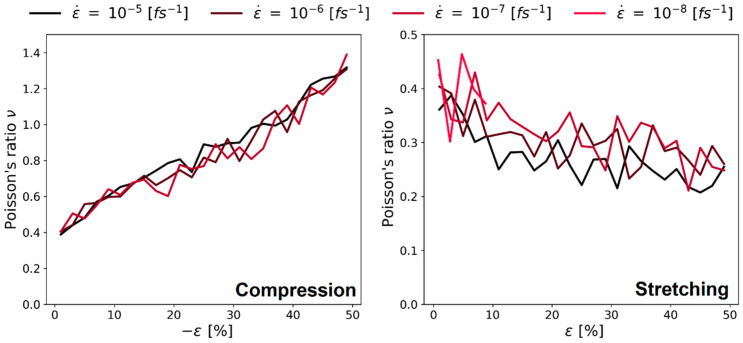
Continuous deformation mode, Poisson’s ratio vs. strain for different $\dot{\varepsilon }$, system C of Kapton^®^. **Left** panel with stretching the system; **right** panel with compression.

During the stretching of the simulation box, the Poisson’s ratio decreased from values around 0.4 down to ν = 0.3 (see right panel Figure 14). Other researchers found the same influence, namely a decrease of the Poisson’s ratio for polystyrene for increasing strain ([31,34]; these observations were made below the glass-transition temperature, the same as in our study). Finally, there was no clear trend on how the Poisson’s ratio changed when changing the strain rate.

The left panel of Figure 14 shows the results during compression simulations. The behavior of increasing Poisson’s ratio did not match the expectations, and values over 0.5 [-] wewre physically not plausible (i.e., volume expansion upon compression). However, in the elastic regime (i.e., small deformation), the values around *ν* = 0.4 were in good agreement with the results with a positive strain.

**Figure 15:**
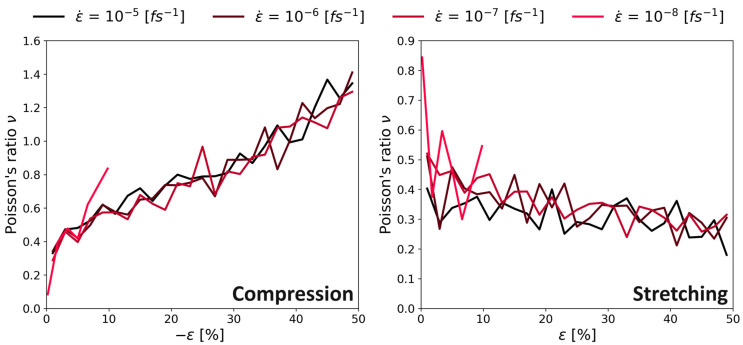
Continuous deformation mode, Poisson’s ratio vs. strain for different $\dot{\varepsilon }$, system C of PMDA-BIA. **Left** panel with stretching the system; **right** panel with compression.

Figure 15 shows the Poisson’s ratio of PMDA-BIA. As the behavior is very similar to the case of Kapton^®^ shown in Figure 14, we assume the same reasons for that behavior as before. Contrary to the investigation of Kapton^®^ the slowest deformation rate ($10^{-8}fs^{-1})$ leads to extremely high Poisson’s ratios and large fluctuations.

**Figure 16:**
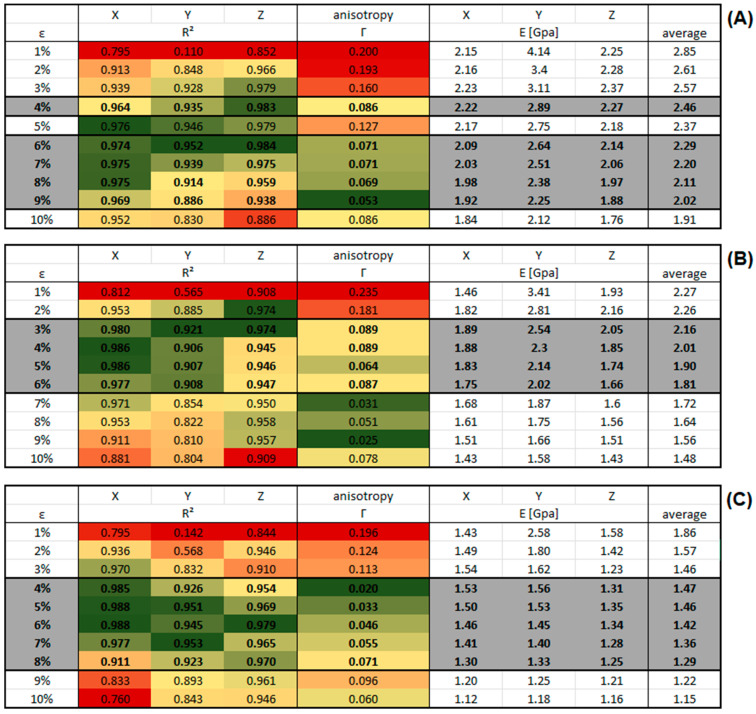
Results tables of Young’s modulus for system C of Kapton in the continuous deformation mode for different strain rates at ambient pressure. The results for R^2^ and the anisotropy Γ were color-coded to indicate the following: green means a high level of matching the criteria, red means a low level of matching the criteria. Rows matching both criteria were highlighted in grey and their values are displayed bold. (**A**) Strain rate of 1 × 10^−6^ (fs^−1^). (**B**) Strain rate of 1 × 10^−7^ (fs^−1^). (**C**) Strain rate of 1 × 10^−8^ (fs^−1^).

Figure 16 shows the result tables for the continuous deformation mode simulations of system C with different deformation rates. The results for R^2^ and the anisotropy Γ were color-coded to indicate the following: green means a high level of matching the criteria, red means a low level of matching the criteria. These criteria were defined as follows: Quality of a linear fit R^2^ > 0.88 in each direction and isotropy parameter Γ < 0.11, rows matching both criteria were highlighted in grey. Finally, we note in passing that our prediction for the spatially averaged Young’s modulus at 4% deformation in the results table (Figure 16A) replicates the value from the literature (2.5 (GPa)) almost perfectly [30].

**Figure 17:**
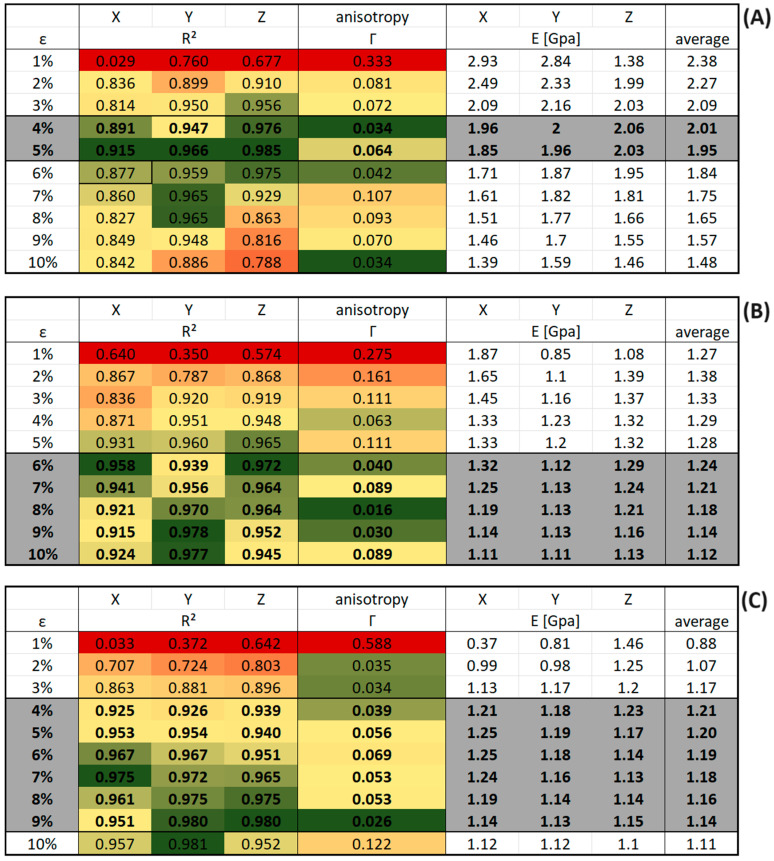
Results tables of Young’s modulus for system C of PMDA-BIA in the continuous deformation mode for different strain rates at ambient pressure. The results for R^2^ and the anisotropy Γ were color-coded to indicate the following: green means a high level of matching the criteria, red means a low level of matching the criteria. Rows matching both criteria were highlighted in grey and their values are displayed bold. (**A**) Strain rate of 1 × 10^−6^ (fs^−1^). (**B**) Strain rate of 1 × 10^−7^ (fs^−1^). (**C**) Strain rate of 1 × 10^−8^ (fs^−1^).

Figure 17 shows the results table for system C of PMDA-BIA under the same criteria as for Kapton^®^. The rows that match both criteria were highlighted in grey as before. Except for the case of a deformation rate of 1 × 10^−6^ $fs^{-1}$ the values that fulfill the criteria are found at higher strains compared to Kapton^®^.

## 4. Conclusions

In our present contribution, a method was described to predict the Young’s modulus of the frequently used polyimide, namely Kapton^®^. Specifically, we used MD simulations with the OPLS-AA force field to represent this popular polyimide. Additionally, the polymer PMDA-BIA was investigated as a second polyimide to get a better understanding of whether our procedure applies to polyimides in general or not.

A preparation procedure that allows for maximum flexibility in terms of using different materials is described. With this preparation method, a polymer can be used and adapted only by changing the geometry of the monomer. This was demonstrated by investigating the polymer PMDA-BIA additional to Kapton^®^, with the same workflow as described in our present work. The Young’s modulus and the Poisson’s ratio of the polymers were determined with two modes for different system sizes, different pressures, and temperatures.

For the investigated system in the relaxation mode, it was found that if the pressure is at 1 (atm) the system is breaking apart and will not have any calculable Young’s modulus in the case of Kapton^®^. A similar behavior was found for PMDA-BIA, although the system did not break apart completely (Young’s modulus at 1 atm was only 30% of the Young’s modulus found at 750 atm). While the relaxation mode was strongly affected by a change in pressure, the continuous deformation mode simulations were strongly affected by the strain rate. The anisotropy Γ and the correlation coefficient R^2^ (determined from a linear approximation of the stress and strain) were introduced to determine the configuration at which the system is most isotropic and behaves linear elastic. Due to this procedure, it is now possible to investigate the influence of different simulation boundary conditions, e.g., pressure and temperature, as objectively as possible. Compared to the literature data, our continuous deformation mode simulation at 10^−6^ (fs^−1^) deformation rate replicated these results best. The Young’s modulus of Kapton^®^ of 2.5 GPa is in perfect agreement with literature values that range from 2.5 GPa [35] to 3.2 GPa [36]. Lei et al. [4] reported a Young’s modulus of PMDA-BIA from a simulation of 5.37 GPa and referenced 8.5 GPa as the experimentally found Young’s modulus of Song et al. [37].

The investigation of the system size showed that—contrary to Ries et al. [31]—the system size has an impact on the results for the polyimides Kapton^®^ and PMDA-BIA. The larger system behaved more isotropic and was less affected by the direction of deformation (both for compression and stretching). For future research in that area, it is important to determine a sufficient system size to ensure an isotropic behavior of the material. This also suggests future investigations regarding the optimal system size, i.e., the sweet spot between a low anisotropy parameter Γ and the computational effort spent.

Regarding the Poisson’s ratio, the continuous deformation mode appears to be the more reliable one. The results of the continuous deformation mode simulations replicated the results from literature within their reported range for Kapton^®^. Both the results for system B with 0.33 and system C with around [35]. However, the results from the relaxation mode simulations underestimate the Poisson’s ratio in comparison to the literature data by 50% for system B. The Poisson’s ratio for system C is about 25% underestimated. Unfortunately, there was no literature data on the Poisson’s ratio for PMDA-BIA. However, the differences between relaxation mode and continuous deformation mode simulations were identical to that for Kapton^®^. Both systems B and C showed a higher Poisson’s ratio in comparison to the simulations of Kapton^®^. Our contribution showed that there is a pressure dependence when performing relaxation mode simulations, as suggested initially by Riccardi et al. [7]. Specifically, we find that for Kapton^®^, a pressure of 750 (atm) showed the best results for the Young’s modulus in comparison with experimental data from the literature. Furthermore, at a pressure of 1 (atm), system B lost its structure and the simulation needed to be conducted at elevated pressures. Therefore, when investigating a homogeneous system, a simulation of the well-established tensile test is the more reliable way to obtain results that should replicate experimental values. In contrast, the relaxation mode is recommended to obtain a locally resolved determination of Young’s modulus and Poisson’s ratio, as described by Riccardi et al. [6,7].

In summary, while Odegard et al. [5] already showed that the OPLS-AA force field can describe a single polyimide in MD simulations, we showed that this force field is also adequate for two other polyimides. This is of critical importance since future research may also be interested in other properties of polyimides (e.g., the diffusion behavior of ions) at different temperatures and molecular structures of the polymer. Since the OPLS-AA force field was built “*… to give highly accurate descriptions of fluids*” [18], we are optimistic that this force field can give a robust and accurate prediction for such applications. In future, this may allow researchers to simulate polyimides with a novel structure under extreme conditions that are difficult to replicate in a physical experiment.

## Figures and Tables

**Table 1:** Different system sizes and chain lengths for PMDA-ODA (Kapton^®^).

System	Chains	Monomers per Chain	Atoms
A	27	5	5265
B	16	20	12,480
C	80	20	62,400

**Table 2:** Different system sizes and chain lengths for PMDA-BIA.

System	Chains	Monomers per Chain	Atoms
B	16	20	14,496
C	80	20	72,480

## Data Availability

The data that support the findings of this study are available upon request from the corresponding author.
